# Effects of Sample Deposition Medium and Drying on Spectroscopic Quantification of Lipid Biomarkers in Respiratory Distress Syndrome

**DOI:** 10.3390/bios16030154

**Published:** 2026-03-10

**Authors:** Zixing (Hings) Luo, Waseem Ahmed, Anthony D. Postle, Ahilanandan Dushianthan, Michael P. W. Grocott, Ganapathy Senthil Murugan

**Affiliations:** 1 Optoelectronics Research Centre, University of Southampton, Southampton SO17 1BJ, UKwaseem.ahmed@soton.ac.uk (W.A.); 2 Perioperative and Critical Care Theme, NIHR Southampton Biomedical Research Centre, University Hospital Southampton NHS Foundation Trust, Southampton SO16 6YD, UK; a.d.postle@soton.ac.uk (A.D.P.); a.dushianthan@soton.ac.uk (A.D.); mike.grocott@soton.ac.uk (M.P.W.G.); 3 Clinical and Experimental Sciences, Faculty of Medicine, University Hospital Southampton NHS Foundation Trust, Southampton SO16 6YD, UK; 4 General Intensive Care Unit, University Hospital Southampton NHS Foundation Trust, Southampton SO16 6YD, UK

**Keywords:** ATR-FTIR, dried-film FTIR spectroscopy, machine learning, neonatal respiratory distress syndrome

## Abstract

Rapid point of care assessment of pulmonary surfactant composition by measuring the lecithin/sphingomyelin (L/S) ratio could improve management of patients with neonatal respiratory distress syndrome (nRDS). Attenuated total reflectance Fourier transform infrared spectroscopy (ATR-FTIR) offers a practical route to making such measurements, but the influence of the sample solvent prior to drying on measurement repeatability is poorly understood. We compare films dried from dichloromethane (DCM) and water (AQ) solvents (DCM-dry route vs. AQ-dry route) by ATR-FTIR and show that spectra from the AQ-dry route increased the signal-to-noise ratio (SNR) of a representative (2920 cm^−1^) absorption peak for the mixture from 20.13 to 128.20 and for human endotracheal aspirate (ETA) from 6.33 to 8.13. A mixed nested analysis of variance (ANOVA) showed that drying route accounted for 89.52% of mixture peak height variance and reduced percent relative standard deviation (%RSD) from 23.5% to 16.2%, corroborated by multivariate analysis for ETA. We further demonstrate that partial least squares regression (PLSR) models trained on AQ-dry mixture spectra predicted L/S (R^2^ = 0.91; root mean square error (RMSE) = 0.31) with 95% prediction interval grey-zone interpretation around L/S = 2.2, complemented by a receiver operating characteristic area under the curve (ROC-AUC) of 0.978.

## 1. Introduction

Neonatal respiratory distress syndrome (nRDS) remains a major cause of morbidity and mortality in preterm infants due to immature lungs and a deficiency or dysfunction of pulmonary surfactant. Surfactant is a lipid–protein complex produced by type II alveolar epithelial cells and is essential for reducing alveolar surface tension to prevent collapse during breathing. Insufficient surfactant quantity, altered composition, or inactivation leads to increased surface tension, impaired gas exchange, and rapid respiratory compromise requiring urgent intervention. Early and accurate assessment of surfactant status is therefore critical for guiding surfactant replacement therapy and improving clinical outcomes in affected neonates [1,2].

Pulmonary surfactant is composed predominantly of phospholipids, mainly phosphatidylcholine and its disaturated form dipalmitoylphosphatidylcholine (DPPC), alongside 1-palmitoyl-2-oleoylphosphatidylcholine (POPC), sphingomyelin (S), phosphatidylglycerol (PG), neutral lipids including cholesterol (Chol), and a smaller but functionally important protein fraction [3,4,5]. The ratio of lecithin (L; primarily DPPC + POPC) to sphingomyelin (L/S ratio) is widely used as a biochemical marker of lung maturity and is strongly associated with nRDS risk [6,7,8]. However, current laboratory-based approaches for quantifying surfactant components—including mass spectrometry [9,10,11,12,13,14], chromatography [15,16,17,18,19], and the enzyme-linked immunosorbent assay (ELISA) [20,21]—are expensive, time-consuming, and unsuitable for point-of-care decision-making in neonatal intensive care.

Vibrational spectroscopy has emerged as a promising alternative for rapid, reagent-free biochemical analysis. Fourier transform infrared (FTIR) spectroscopy, particularly when combined with attenuated total reflectance (ATR) sampling, enables direct measurement of surfactant components with minimal sample preparation. Recent studies have demonstrated the feasibility of using ATR-FTIR with machine learning to predict surfactant lipid concentrations and estimate L/S ratios [22,23,24]. However, translation toward clinically deployable workflows remains constrained by two practical issues: (i) preparation-dependent film formation can strongly influence spectral quality and quantitative repeatability, and (ii) predictive uncertainty is not always propagated to decision-level interpretation around diagnostic thresholds.

These limitations are especially relevant because clinical respiratory specimens are aqueous and contain matrix interferents (notably proteins and salts) that can confound lipid quantification. Clinically aligned workflows, therefore, typically enrich the lipid fraction prior to ATR-FTIR measurement and analyse it as a dried film to mitigate strong water absorption and enhance lipid spectral features. In this setting, the liquid used to deposit the lipid fraction prior to drying becomes a key experimental variable. Previous studies have shown that spectroscopic sensing is sensitive to physiochemical influences on the sample interface and it is in this vein that this study seeks to understand where the greatest source of variance to control in a spectroscopy-based point of care measurement device might manifest [25,26,27]. For clarity, throughout this paper, “solvent” refers to the liquid deposition medium and includes both organic solvents and water. Solvent handling and drying can confound quantitative readout by (i) residual deposition medium that distorts baselines and masks diagnostically relevant bands and (ii) drying-induced nonuniformity (e.g., edge enrichment/coffee-ring deposition and thickness gradients) that alters effective sampling of the ATR sensing region, increasing run-to-run variability and reducing the robustness of downstream machine learning models. Rapid evaporation from an organic solvent such as dichloromethane (DCM) may promote flow-driven redistribution during drying, whereas aqueous resuspension may promote lipid self-assembly prior to deposition and vacuum drying provides a controlled removal step. Together, these considerations motivate the hypothesis that an aqueous resuspension route can improve film formation and reduce preparation-dependent dispersion under otherwise identical measurement conditions.

In the present study, the analytical objective was to quantify how the deposition medium used for the lipid fraction (and the resulting dried-film formation) influences spectral quality and repeatability in dried-film ATR-FTIR. We compare two dried-film preparation routes—vacuum drying from DCM (DCM-dry) and vacuum drying after aqueous resuspension (AQ-dry)—and evaluate route effects using complementary measures of spectral quality and film morphology. Repeatability is quantified using a structured nested replicate design and variance component decomposition by mixed nested analysis of variance (ANOVA), separating route effects from run-level and scan-level variability. To assess feasibility in a clinically relevant matrix under the same workflow, a lipid extract prepared from undiluted human endotracheal aspirate (ETA) is analysed using both routes. Finally, informed by the drying route comparison, partial least squares regression (PLSR) models are developed for multicomponent lipid quantification, and predictive uncertainty is propagated to L/S estimation using prediction intervals and a grey-zone decision framework around a clinically used L/S threshold; classification performance is additionally summarised using receiver operating characteristic (ROC) analysis as a threshold-independent metric.

## 2. Materials and Methods

### 2.1. Preparation of Individual Lipid Standards, Five-Lipid Mixtures and Human ETA Sample

A synthetic lung surfactant model comprising five major lipid components—DPPC (Avanti Research, Alabaster, AL, USA), POPC (Avanti Research, Alabaster, AL, USA), S (Merck KGaA, Darmstadt, Germany), PG (Avanti Research, Alabaster, AL, USA) and Chol (Merck KGaA, Darmstadt, Germany)—was prepared to generate a physiologically relevant multicomponent system. Purified powdered lipids were dissolved in DCM (Fisher Scientific, Loughborough, UK) and methanol (Fisher Scientific, Loughborough, UK) to produce stock solutions. Methanol was added solely to aid solubility and was removed prior to measurement by drying samples at 63 °C under a nitrogen stream. Each mixture was redissolved in 1 mL of DCM or resuspended in 1 mL of distilled water and vortex-mixed until uniformly turbid. Individual lipid standards (1 mM) were prepared in parallel for spectral inspection and preparation route comparison.

Final lipid concentrations were defined using an Extreme Vertices mixture design, following the approach of Ahmed et al. and constrained within physiologically relevant ranges [24]. Concentration ranges included: DPPC (1.035–1.308 mM), POPC (0.508–0.632 mM), S (0.273–0.853 mM), PG (0.008–0.333 mM) and Chol (0.207–0.310 mM). These bounds were retained from an established five-lipid calibration domain to preserve an identical mixture design space while comparing preparation routes. This enables observed differences between the dried-film preparation routes to be attributed to preparation-dependent film formation and repeatability, rather than to changes in mixture constraints or sampling of a different compositional domain.

In addition to synthetic standards and mixtures, a lipid extract prepared from undiluted human ETA was analysed to assess the feasibility of the synthetic lipid mixture model to quantify the presence of lipid biomarkers in a clinically relevant matrix. ETA lipids were extracted using DCM–methanol–water liquid–liquid extraction based on a modified Folch and Bligh–Dyer approach [28,29,30], to extract the phospholipid fraction and reduce other interferents. The extract was processed through the same two preparation routes mentioned above prior to dried-film ATR-FTIR measurement (Section 2.2 and Section 2.3).

### 2.2. Dried-Film Formation and ATR-FTIR Spectra Acquisition

ATR-FTIR spectra were acquired using a ConcentratIR2™ 23-reflection extended-pathlength silicon ATR element (Specac, Inc., Mount Kisco, NY, USA) coupled to an Agilent Cary 670 FTIR spectrometer (Agilent Technologies, Santa Clara, CA, USA) fitted with a mercury cadmium telluride (MCT) detector. For all measurements, 10 µL of sample was deposited onto the ATR crystal and dried under vacuum for 9 min to form a thin film prior to spectral acquisition. Two dried-film preparation routes were investigated: films deposited from DCM (DCM-dry) and films deposited from aqueous suspension (AQ-dry). In both cases, vacuum drying was performed using a custom vacuum setup to remove the solvent and stabilise the deposited film prior to spectral collection [31]. Vacuum drying was selected to accelerate solvent removal at near-ambient temperature, thereby minimising thermal perturbation of lipid films while improving drying consistency for quantitative ATR-FTIR spectra acquisition.

Following drying, nine consecutive spectra were collected per sample at 4 cm^−1^ resolution with 32 scans under nitrogen purge. Microscope images of the dried films were acquired using a Dino-Lite 5MP Edge AM7915MZT digital microscope (AnMo Electronics Corporation, New Taipei City, Taiwan) at 51.7× magnification following spectral collection. Unless otherwise stated, samples were measured in triplicate (three independently prepared films per route; nine spectra per film). For the nested repeatability studies reported in Section 3, nine independently prepared films (‘runs’) were acquired per route, with nine spectra per run. A fresh background spectrum was recorded prior to each set. The ATR crystal was cleaned between measurements using methanol/DCM (1:9, *v*/*v*) followed by DCM, and surface cleanliness was confirmed by the absence of residual lipid bands (peak absorbance < 0.0004) in the spectrum.

### 2.3. Spectral Quality Metrics and Repeatability Assessment

To quantify preparation-dependent spectral quality, the most intense peak with the highest signal-to-noise ratio (SNR) across the tested lipid systems was used, identified as the methylene asymmetric stretching band at 2920 cm^−1^. While the other band could have been used, the relative peak height compared to the instrument noise would have been reduced and it was decided to analyse the best case. For each spectrum, the peak height was extracted from baseline-corrected absorbance spectra using Peak^®^ Spectroscopy Software version 4.00.484 (Operant LLC, Burke, VA, USA). Spectral noise was quantified using the root mean square (RMS) noise value reported by the software within a fixed-feature minimal-baseline region (2200–2000 cm^−1^), applied consistently across all spectra. Peak SNR was defined as the ratio of 2920 cm^−1^ peak height to RMS noise. Peak SNR values were computed for each spectrum and summarised per sample type and preparation route to provide a consistent comparison of spectral quality between DCM-dry and AQ-dry films.

Dried-film morphology was assessed qualitatively using optical microscope images acquired after spectral collection. Images were captured under consistent imaging conditions to compare film coverage and deposition patterns between preparation routes, and were used to support the interpretation of preparation-driven changes in spectral quality (e.g., evidence of edge enrichment consistent with coffee-ring drying versus more uniform coverage).

Preparation-dependent repeatability was assessed via the 2920 cm^−1^ peak height rather than the SNR to decouple film deposition variability from variance introduced by the RMS noise estimate. Repeatability was assessed at two levels: (i) between independently prepared films (‘runs’) and (ii) within a single film (nine consecutive spectra per run). A two-factor mixed nested ANOVA was applied with drying route (DCM-dry vs. AQ-dry) treated as a fixed factor and sample run treated as a random factor nested within the route (nine runs per route; nine spectra per run), with within-run variability captured by the residual term [32,33]. Variance components (σ^2^) were estimated for each source term and expressed as variance fractions (%) to provide a variance decomposition view of repeatability. To provide a total route precision that is independent of ANOVA significance testing, relative standard deviation (%RSD) was calculated for the 2920 cm^−1^ peak height within each route as 100 × (standard deviation/mean), using all spectra acquired under that route (*n* = 81 spectra per route for the nested studies).

### 2.4. Spectral Preprocessing and Machine Learning

For machine learning model development, all spectral preprocessing and modelling were performed using Peak^®^ Spectroscopy Software, Python 3.12, and Jupyter Notebook 7.0.8 [23,24]. Raw spectra were baseline-offset at 2800 cm^−1^, truncated to 4000–850 cm^−1^, and smoothed using a Savitzky–Golay 15-point, 3rd order, 2nd derivative filter to reduce noise while preserving peak features [34,35]. To prevent data leakage, training (80%) and test (20%) sets were partitioned by sample rather than by individual spectra.

PLSR models were developed for each individual lipid, as well as for L, which was defined as the combined concentration of DPPC and POPC. This definition reflects the biochemical composition of pulmonary surfactant, where DPPC and POPC constitute the dominant phosphatidylcholine species and together represent the clinically relevant L fraction underpinning the traditional L/S ratio used for foetal lung maturity assessment. The optimal number of latent variables (LVs) was identified using k-fold cross-validation by minimising mean square error (MSE) or maximising mean R^2^ [36]. Final models were retrained on the full training set and evaluated using the independent test set to assess model generalisability.

To quantify predictive uncertainty, 95% prediction intervals were generated using the jackknife+ -after-bootstrap method implemented in the MAPIE Python library version 0.5.0 [37]. This distribution-free approach combines bootstrap resampling with jackknife+ aggregation to produce valid, model-agnostic interval estimates under the assumption of exchangeable data. The method avoids restrictive assumptions of homoscedasticity or Gaussian residuals and produces narrower, more practically interpretable intervals than classical bootstrap-based schemes. Prediction intervals were computed for each lipid model and subsequently propagated to the derived L/S ratio by combining the L and S interval bounds.

Building on the construction of prediction intervals, their diagnostic implications were evaluated by incorporating a clinically relevant grey zone around the established L/S diagnostic cut-off. To reflect uncertainty around the threshold of 2.2, a diagnostic grey zone was defined using the 95% prediction intervals obtained from the lipid concentration models. Predictions with intervals spanning the threshold were assigned to the grey zone, while those entirely above or below it were classified as positive or negative, respectively. Published reference L/S ratios from a neonatal cohort [22] were then used to demonstrate the decision implications of the grey-zone decision rule under the assumed uncertainty model. Infants whose reference L/S ratios fell within the grey zone (defined using the averaged test-set L/S uncertainty) were treated as indeterminate (‘test failures’) under an intention-to-diagnose rule. For reference-positive infants (RDS present), indeterminate predictions were counted as false negatives; for reference-negative infants, they were counted as false positives. Sensitivity and specificity were calculated as follows [38]:

$$Sensitivity=\frac{number\;of\;true\;positives}{number\;of\;true\;positives+number\;of\;false\;negatives}$$

$$Specificity=\frac{number\;of\;true\;negatives}{number\;of\;true\;negatives+number\;of\;false\;positives}$$

ROC analysis was used to quantify threshold-independent discrimination on the synthetic mixture dataset. Binary reference labels were assigned using the true L/S ratio relative to the clinical threshold (L/S = 2.2), and the predicted L/S point estimate was used as the continuous score. The ROC curve was generated by sweeping the decision threshold across the score range, and the area under the curve (AUC) was computed by trapezoidal integration.

## 3. Results and Discussion

### 3.1. Effect of Solvent Origin on Dried-Film Spectral Quality and Repeatability

DCM enables preparation of homogeneous lipid mixtures at controlled composition and concentration and therefore provides a practical medium for generating reproducible calibration standards. Using the dried-film ATR-FTIR workflow described in Section 2, we quantified how the deposition medium (DCM-dry vs. AQ-dry) influences spectral quality and repeatability under identical instrumental conditions. Feasibility in a clinically relevant matrix was additionally assessed using a lipid extract prepared from undiluted human ETA and processed via both routes.

**Figure 1:**
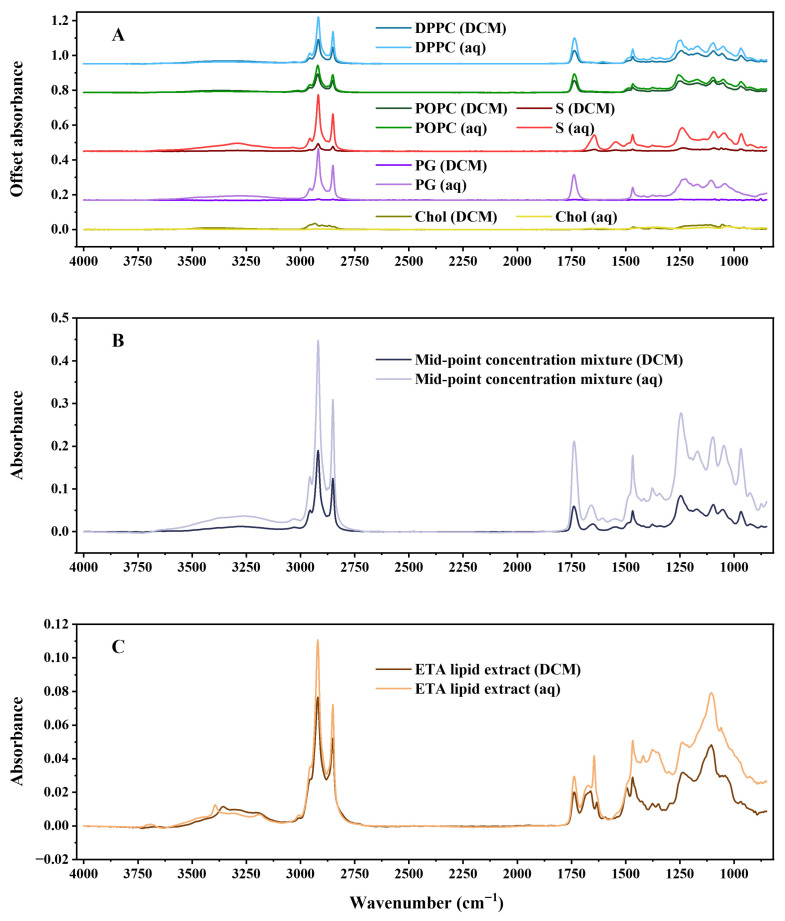
Representative ATR-FTIR spectra acquired on the ConcentratIR2™ 23-reflection silicon ATR element with an MCT detector under identical acquisition settings, comparing DCM-dry and AQ-dry films. (**A**) Individual surfactant lipids (DPPC, POPC, S, PG and Chol). (**B**) Representative mid-point composition five-lipid mixture within the PLSR concentration domain. (**C**) Lipid extract prepared from ETA sample, comparing DCM-dry and AQ-dry preparations. Spectra illustrate preparation-dependent differences in baseline stability and spectral noise, with improved band definition for AQ-dry, particularly in the CH-stretch region (3000–2800 cm^−1^). CO_2_ absorption regions were removed.

Representative ATR-FTIR spectra comparing the two dried-film preparation routes are presented in Figure 1. Spectra were acquired on the ConcentratIR2™ 23-reflection silicon ATR element with an MCT detector under identical acquisition settings and are shown for films dried from DCM and aqueous origin. Figure 1A shows the five individual surfactant lipids in a concentration of 1 mM, confirming that the expected lipid features are preserved under both routes while highlighting systematic differences in baseline stability and spectral noise that are consistent with preparation-dependent film formation. Selected wavenumber regions associated with key vibrational bands in the tested surfactant lipids are summarised in Table 1 [39,40,41,42,43,44,45,46]. DPPC and POPC both display methylene stretching bands at 2950–2850 cm^−1^ and a clear ester carbonyl band centred at 1740–1735 cm^−1^, consistent with acyl-chain and ester linkages in phosphatidylcholine. S is distinguished by a strong amide I band near 1650–1645 cm^−1^ (with a weaker amide II contribution at 1540 cm^−1^), reflecting the amide linkage within the sphingolipid (ceramide) backbone. A pronounced band at 1260–1240 cm^−1^ is observed for all phospholipids (DPPC, POPC, PG and S) but is absent in Chol; this feature is assigned to the asymmetric stretching vibration of the phosphate-ester (PO_2_^−^) group in phospholipid headgroups and is readily observed in the dried-film spectra, where solvent absorption does not dominate this region. Chol, lacking a phosphate headgroup, shows no 1260–1240 cm^−1^ band and remains most readily identified by its characteristic fingerprint in the 3000–2800 cm^−1^ region and unique low-wavenumber features.

Figure 1B focuses on a mid-point concentration mixture, selected because its composition lies close to the centre of the concentration ranges used for PLSR model development and therefore provides a representative mid-point case for assessing preparation effects under mixture conditions (1.172 mM DPPC, 0.570 mM POPC, 0.688 mM S, 0.165 mM PG, and 0.259 mM Chol). For this mixture, the AQ-dry spectra show clearer band definition and reduced baseline fluctuation, particularly in the CH-stretch region (3000–2800 cm^−1^), whereas DCM-dry spectra exhibit greater spectral variability across repeated measurements. Figure 1C shows the lipid extract prepared from an undiluted ETA sample, comparing films generated via DCM-dry and AQ-dry preparation. In this representative clinical matrix-derived extract, both routes yield interpretable lipid features, and the AQ-dry preparation provides better band definition in the CH-stretch region, supporting compatibility with post-extraction aqueous resuspension film preparation for clinical lipid extracts.

To quantify these preparation-dependent differences in a simple and consistent manner, spectral quality was assessed using the 2920 cm^−1^ peak SNR. Repeatability was evaluated separately for the mid-point concentration mixture and for the ETA lipid extract using an identical nested replicate design (nine independently prepared films per route and nine spectra per film) with nested variance analysis. Importantly, while this section uses the 2920 cm^−1^ band as a practical quality metric, the subsequent PLSR models were trained on complete spectral profiles (4000–850 cm^−1^) rather than single-band predictors.

**Figure 2:**
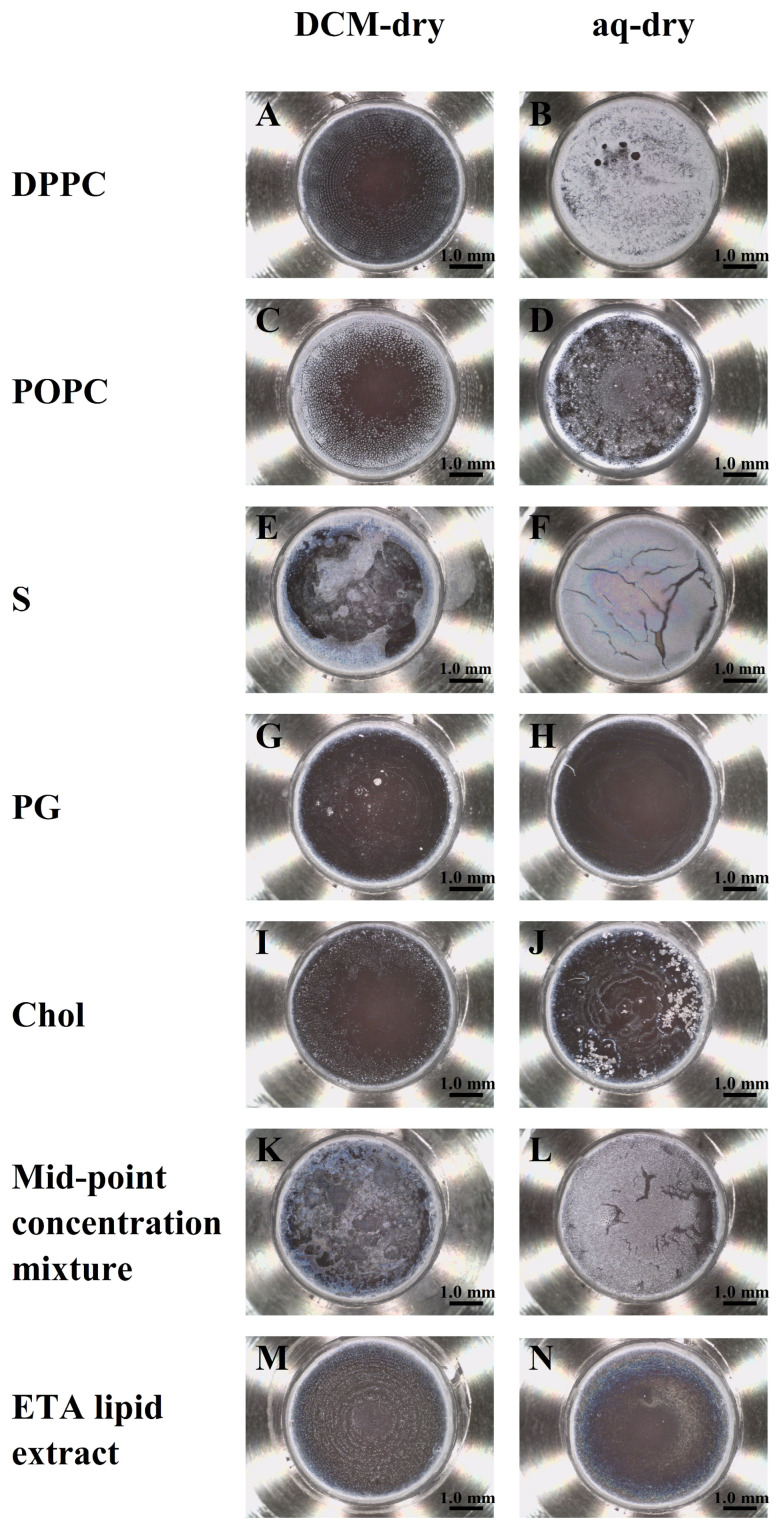
Representative microscope images of vacuum-dried films formed on the ConcentratIR2™ ATR crystal following preparation from DCM dissolution (DCM-dry; left column) and aqueous resuspension (AQ-dry; right column). Images are shown for DPPC (**A**,**B**), POPC (**C**,**D**), S (**E**,**F**), PG (**G**,**H**), Chol (**I**,**J**), mid-point concentration mixture (**K**,**L**) and ETA lipid extract (**M**,**N**). Scale bars: 1.0 mm.

Table 2 summarises the 2920 cm^−1^ peak SNR for DCM-dry and AQ-dry preparations across the seven sample types. The AQ-dry samples showed an increased SNR for DPPC (45.187 to 71.284), S (6.810 to 102.326), PG (0.975 to 40.872) and the mid-point concentration mixture (20.128 to 128.200), while POPC was essentially unchanged (20.405 to 20.548). The ETA lipid extract also showed a modest SNR increase under AQ-dry (6.334 to 8.134), supporting the compatibility of the AQ-dry route applied to a clinical lipid extract. In contrast, Chol exhibited substantially lower SNR under AQ-dry (3.679 to 0.062), consistent with poor dispersion/phase separation when deposited as a single component via an aqueous route. These trends are supported by the corresponding microscope images (Figure 2; scale bars: 1.0 mm), which show preparation-dependent differences in dried-film morphology consistent with differences in effective coverage and film uniformity, which can influence coupling to the ATR evanescent field and therefore the observed peak intensities.

For several samples, DCM-dry films exhibit clear evidence of non-uniform deposition and edge enrichment, consistent with a coffee-ring drying pattern (e.g., DPPC, Figure 2A; S, Figure 2E; mid-point concentration mixture, Figure 2K). Such annular deposition produces thickness gradients and incomplete/heterogeneous coverage across the sensing area, which can increase low-frequency baseline variation and elevate the root mean square (RMS) noise used in SNR calculation, thereby reducing the measured 2920 cm^−1^ SNR. In comparison, AQ-dry films (except Chol) appear more evenly distributed across the ATR crystal for the synthetic samples, consistent with the higher SNR values reported in Table 2. POPC shows broadly comparable deposition between routes (Figure 2C,D), in agreement with the minimal SNR change. Chol represents a distinct case—the AQ-dry film (Figure 2J) shows pronounced phase separation/particulate domains and strong edge features, consistent with poor dispersion when Chol is deposited as a single component via an aqueous route. This morphology is expected to reduce the effective and spatially uniform contact area with the ATR sensing region (lowering the coupled lipid signals) and might increase local thickness heterogeneity/scattering (increasing the background variability captured by the RMS noise term), thereby lowering the 2920 cm^−1^ peak SNR. For the ETA lipid extract (Figure 2M,N), both routes yield continuous films, but with distinct millimetre-scale drying patterns: the DCM-dry film (Figure 2M) appears comparatively uniform with only weak concentric drying striations, whereas the AQ-dry film (Figure 2N) shows a predominantly uniform interior with mild peripheral edge enrichment (coffee-ring) and subtle interference colouration, indicative of some thickness gradients. These observations highlight that clinical matrix-derived extracts can exhibit more complex drying behaviour than synthetic lipid samples; however, the improved repeatability observed for AQ-dry indicates that these visible millimetre-scale drying features did not compromise the ATR-FTIR readout under our measurement conditions.

Taken together, Table 2 and Figure 2 indicate that the drying route can alter both the spectral SNR and film morphology, implying that preparation-dependent deposition effects may be a dominant source of measurement variability. Because diagnostic modelling ultimately depends on the reproducibility of spectra obtained from multicomponent surfactant mixtures, repeatability was quantified for the mid-point concentration mixture, which represents a mid-point composition within the PLSR concentration domain and was measured with a structured replicate design (nine independently prepared films per route; nine spectra per film). An equivalent structured replicate design was also applied to the ETA lipid extract to quantify route-dependent repeatability in a clinically relevant matrix. This replicate structure enables variance decomposition by nested ANOVA to quantify the relative contributions of preparation route, measurement-to-measurement variability and scan-level noise, and to determine whether the SNR improvements observed for AQ-dry translate into improved repeatability under mixture and clinical extract conditions.

To evaluate preparation-dependent repeatability under mixture conditions, the mid-point concentration mixture was selected for a nested replicate study (nine independent runs per drying route; nine spectra acquired per run). Repeatability was assessed using the 2920 cm^−1^ peak height, which provides a direct measure of CH_2_ band intensity without incorporating the RMS noise term used for the SNR. Variance decomposition of the nested replicate structure indicated that the drying route accounted for 89.52% of the total variance at the 2920 cm^−1^ peak height (Cohen’s d (|d|) = 1.14), while run-to-run variability nested within the route accounted for 10.48%; the residual (scan-to-scan) contribution was negligible (1.36 × 10^−4^%) (Table 3). A two-factor mixed nested ANOVA was applied, with the drying route treated as a fixed factor and sample run treated as a random factor nested within the drying route [32,33]. Consistent with the variance decomposition, the drying route (DCM-dry vs. AQ-dry) produced a significant effect on the 2920 cm^−1^ peak height (F = 77.857, *p* = 1.521 × 10^−7^), while run-to-run differences within each route were also significant when tested against the within-run residual (F = 692,177, *p* < 5 × 10^−324^) (Table 3). The residual term was several orders of magnitude smaller than the run-level component, indicating that scan-to-scan variability was negligible relative to film preparation variability. To provide a route-specific measure of practical repeatability, %RSD was also calculated across all spectra within each route. For the mid-point concentration mixture, %RSD decreased from 23.5% for DCM-dry to 16.2% for AQ-dry, consistent with improved preparation repeatability under mixture conditions. Route-stratified within-route variance components were estimated for the same endpoint (2920 cm^−1^ peak height): σ^2^_DCM-dry_ = 2.44 × 10^−3^ and σ^2^_AQ-dry_ = 6.75 × 10^−3^. Despite the lower absolute dispersion under DCM-dry, the mean peak height under DCM-dry was substantially lower (0.210) than under AQ-dry (0.507). Consequently, when precision is expressed relative to the mean, %RSD is higher for DCM-dry even though σ^2^ is lower. This reconciles the apparent discrepancy between ‘better σ^2^’ and ‘worse %RSD’ for the mid-point concentration mixture and is also consistent with the observation of a poorer SNR under DCM-dry, which may reflect a reduced effective signal and/or increased baseline/noise contributions relative to AQ-dry.

An equivalent nested analysis was performed for the ETA lipid extract to assess whether the same preparation-dependent variance structure is observed in a clinically relevant matrix (Table 4). In contrast to the mid-point concentration mixture, the drying route contributed 0% of the total variance for the ETA extract (F = 0.523, *p* = 0.480, |d| = 0.057), whereas run-to-run variability nested within the route accounted for 99.97% of the variance; the residual contribution remained small (0.03%). Notably, despite the run-dominated variance structure for peak height, AQ-dry maintained—and modestly improved—spectral quality for the ETA extract, increasing the 2920 cm^−1^ peak SNR from 6.334 (DCM-dry) to 8.134 (AQ-dry) (Table 2), supporting the practical capability of the AQ-dry route for clinical matrix-derived lipid extracts. For the ETA lipid extract, %RSD likewise decreased from 30.2% for DCM-dry to 11.2% for AQ-dry, indicating improved repeatability even though the route’s effect on mean 2920 cm^−1^ peak height was not statistically significant. The route-specific means were similar (DCM-dry: 0.114; AQ-dry: 0.105), consistent with the non-significant route fixed effect (*p* = 0.480). Although the mean of the peak height is not significantly different, the variance component of the AQ-dry route is ~9-fold lower than that of the DCM-dry route (σ^2^_AQ-dry_ = 1.36 × 10^−4^ vs. σ^2^_DCM-dry_ = 1.18 × 10^−3^), further showing markedly better repeatability of the AQ-dry route. Accordingly, for the ETA matrix, the deposition medium primarily affects precision (run-to-run deposition variability and/or noise structure) rather than producing a systematic shift in mean peak height. Together, these results indicate that, for the ETA extract, AQ-dry improves practical precision and spectral quality without introducing a systematic shift in mean peak height, while overall variability remains dominated by run-dependent deposition in a complex matrix. This supports the feasibility of aqueous resuspension prior to dried-film ATR-FTIR for ETA-derived lipid extracts, with transferability primarily limited by run-dependent deposition variability rather than route-driven mean shifts. As a robustness check of using the 2920 cm^−1^ peak height, analysis on the 2945 cm^−1^ to 2880 cm^−1^ peak area, and 1740 cm^−1^ carbonyl peak height, gave the same qualitative trends in SNR and variance structure (Supplementary Tables S1–S6).

This variance structure is consistent with dried-film ATR measurements, in which the measured peak height depends not only on composition but also on film morphology and effective coverage across the ATR sensing region. In ATR-FTIR, the measured spectrum arises from a sample interacting with the evanescent field at the point of light undergoing total internal reflection at a sampling interface. Therefore, the nature of the sample’s contact with that interface, the orientation of the molecules with respect to it, the homogeneity or the spatial distribution, and organisation of the sample will all have an impact on the measured spectrum. Sample presentation therefore contributes to the signal intensity and repeatability of the spectrum.

Drying concentrates the sample and removes solvent contribution to the spectrum, which can obscure spectral features and reduce the SNR but, simultaneously, it also impacts how it is deposited and distributed across the crystal. With DCM, rapid drying can give rise to concentration and surface-tension gradients, producing contact line pinning and Marangoni flow, resulting in the deposition of sample components towards the perimeter (coffee-ring deposition), which is more pronounced than in water. In DCM, the lipids are molecularly dispersed, so drying can result in component separation, whereas in water, there is a tendency to form vesicles—which are assumed to be homogenous—so drying causes the aggregation and collapse of these structures, which leads to a comparatively more uniform and thicker film deposition. As the solvent approaches saturation, phase segregation and even crystallisation may result in further sample heterogeneity, introducing scattering and inhomogeneity in the film, both of which can contribute to lower SNR and spectral variance. The ATR-FTIR signal also depends on the orientation of the molecular dipoles in the film relative to the evanescent field. While no conclusions can be drawn from this study on the orientation of the molecules, this may also have contributed to the increased signal observed from the water-dried samples. For the mid-point concentration mixture, the dominance of the drying-route variance fraction and the negligible scan-level term demonstrate that AQ-dry provides substantially improved preparation-dependent repeatability under mixture conditions, supporting its use for subsequent PLSR model development. For the ETA lipid extract, the near-zero route variance fraction indicates that variability is dominated by run-dependent deposition effects within a complex matrix, even though interpretable lipid spectra are obtained. The mean peak height is strongly route-dependent for the mid-point concentration mixture (0.210 vs. 0.507), suggesting that the drying route changes the average effective signal in this controlled mixture, whereas for the ETA extract, the mean is similar between routes (0.114 vs. 0.105) and route effects are expressed mainly as differences in variability and spectral quality (σ^2^, %RSD, and SNR).

**Figure 3:**
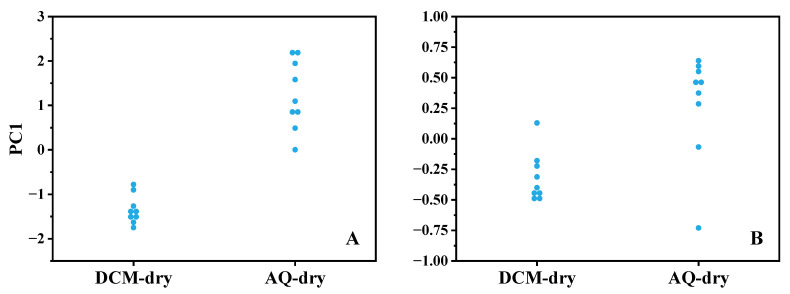
PC analysis of ATR-FTIR spectra for samples prepared by different drying routes. PC1 scores for DCM-dry and AQ-dry preparations are shown for the mid-range mixture (**A**) and human ETA extract sample (**B**).

The study of the impact of drying route extended the univariate approach to a principal component (PC) analysis as an alternative (and multivariate) metric to establish whether the findings of the univariate analysis were dependent on the use of the peak at 2920 cm^−1^, or whether this was reflected more broadly in the spectrum. The first principal component variance was independently considered (mid-range and human ETA extract samples) and demonstrates that the dominant variance axis corresponds to route separation, which is consistent with film morphology dependence on solvent (|d|: mid-range mixture = 4.3, ETA sample = 1.7) (Figure 3). The multivariate analysis uses the whole spectrum and corroborates the key finding of the univariate analysis of the 2920 cm^−1^ peak height that the drying route dominates variance in the mid-range sample, even in the human ETA sample’s case.

This study examined synthetic lipid mixtures at expected physiological concentrations and lipids extracted from a human ETA sample, which are compositionally distinct sources. The PC analysis demonstrated that, in both cases, the dominant variance axis aligned with the drying route, which was consistent with preparation-dependent film morphology rather than composition-specific factors. This observation—while not conclusive—lends confidence to the general applicability of these findings when considering similar lipid extracted fluids from the bronchoalveolar lavage (BAL) or sputum. More complex samples, such as “neat” BAL or ETA, which have additional protein and carbohydrate components, would likely manifest as increased preparation-dependent film heterogeneity, leading primarily to increased spectral variance and reduced SNR.

### 3.2. Predictive Machine Learning Model Development for Water-Based Vacuum-Dried Films

**Figure 4:**
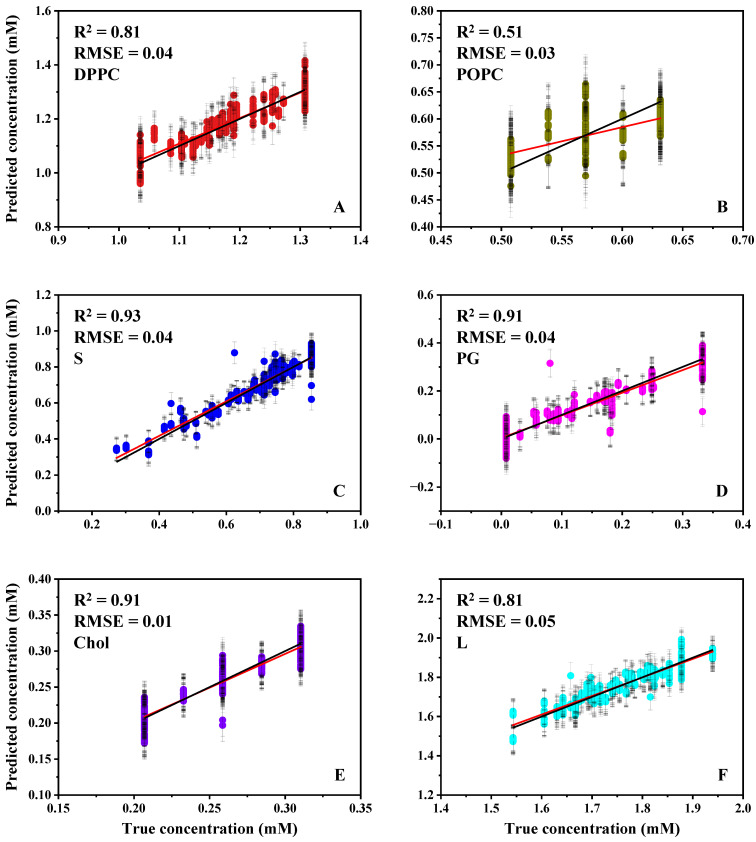
Predicted vs. true concentrations of the water-based complex mixture model of DPPC (**A**), POPC (**B**), S (**C**), PG (**D**), Chol (**E**) and L (total concentration of DPPC and POPC, (**F**)) with the R^2^ and RMSE values for each lipid. The red line in each graph indicates the linear fitting curve, while the black line corresponds to the expected prediction value (y = x). The 95% prediction intervals were generated using the jackknife+ -after-bootstrap method and are indicated by the error bars.

On the basis of the preparation route comparison in Section 3.1, the aqueous resuspension followed by vacuum drying (AQ-dry) protocol was adopted for predictive model development. Five-component lipid mixtures prepared in an aqueous matrix were deposited as 10 µL aliquots onto the ATR crystal and vacuum-dried to form thin films before spectroscopic measurement. Predicted versus true concentrations for the resulting PLSR models are presented in Figure 4. The S model achieved strong predictive performance (R^2^ = 0.93, RMSE = 0.04), supporting reliable estimation of the S component under these measurement conditions. DPPC and POPC were additionally reported both individually and as a combined lecithin metric, L (DPPC + POPC), reflecting the clinical relevance of total phosphatidylcholine in lung surfactant and providing a compositionally meaningful predictor in the presence of spectral overlap between the two species. In the present dataset, L performed comparably to DPPC (L: R^2^ = 0.81, RMSE = 0.05; DPPC: R^2^ = 0.81, RMSE = 0.04), whereas POPC alone remained less predictable (R^2^ = 0.51, RMSE = 0.03), consistent with shared CH-stretch and carbonyl features across phosphatidylcholine species in multicomponent mixtures. PG and Chol are also well predicted within the tested range (PG: R^2^ = 0.91, RMSE = 0.04; Chol: R^2^ = 0.91, RMSE = 0.01). Notably, although single-component Chol exhibited a reduced SNR under AQ-dry (Table 2), Chol was well predicted within multicomponent mixtures, consistent with co-deposition alongside phospholipids, yielding a stable multivariate signature. Collectively, these results indicate that the AQ-dry workflow enables accurate multivariate quantification of the principal diagnostic components required for downstream L/S estimation, while highlighting residual limitations in separating closely related phosphatidylcholine species within complex mixtures.

**Figure 5:**
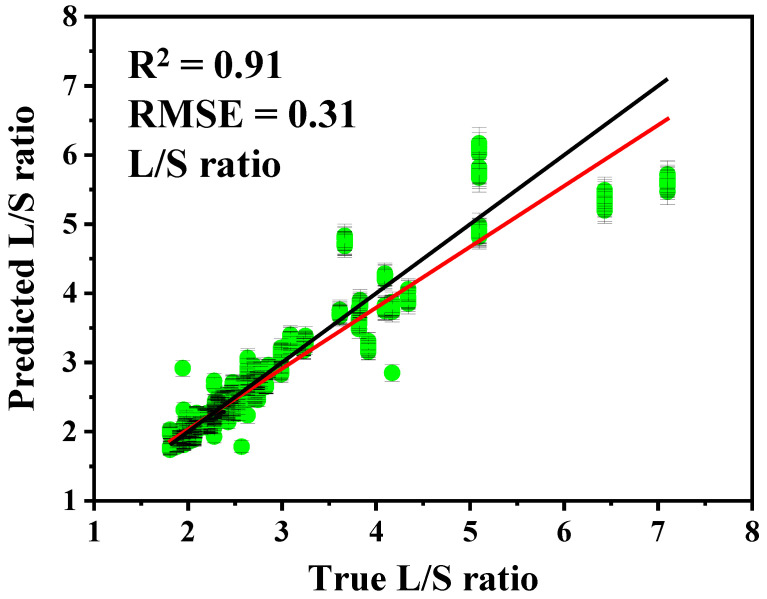
The predicted L/S ratios based on the L and S models generated from water-based synthetic complex mixture with the R^2^ and RMSE values. The red line indicates the linear fitting curve, while the black line corresponds to the expected prediction value (y = x). The 95% prediction intervals were generated using the jackknife+ -after-bootstrap method and are indicated by the error bars.

On the basis of the individually trained L and S models, the L/S ratio was calculated for each mixture sample and compared with the expected ratio (Figure 5). The derived L/S estimates demonstrated strong correspondence with the expected values (R^2^ = 0.91, RMSE = 0.31), indicating that the water-based vacuum-dried workflow supports reliable ratio estimation from the component predictions. As the L/S ratio is intended for downstream diagnostic interpretation, performance was assessed not only using point-estimate metrics but also with explicit quantification of predictive uncertainty. Accordingly, 95% prediction intervals were generated using the jackknife+ -after-bootstrap method and are shown in Figure 5. Prediction intervals for the derived L/S ratio were obtained by propagating the component-level prediction uncertainties from the independently trained L and S models, following our previously reported approach; specifically, the larger of the upper and lower 95% prediction interval half-widths for each component was used as a symmetric uncertainty term for ratio uncertainty propagation [24]. These intervals are carried forward to evaluate robustness and to support decision-relevant interpretation around clinically meaningful L/S thresholds.

### 3.3. Prediction Intervals, Diagnostic Grey Zone Analysis and ROC Performance

In addition to point-estimate metrics (R^2^ and RMSE), predictive uncertainty was evaluated using 95% prediction intervals generated with the jackknife+ -after-bootstrap method. Table 5 summarises the maximum upper and lower prediction intervals for the water-based vacuum-dried models, providing a conservative estimate of uncertainty across the concentration domain. Across all lipid models, the maximum prediction intervals were within ±0.027–0.099 mM, indicating relatively narrow uncertainty bands under the water-based workflow. To translate these interval estimates into a decision-relevant interpretation, uncertainty was next evaluated in the context of the clinically used L/S threshold.

**Figure 6:**
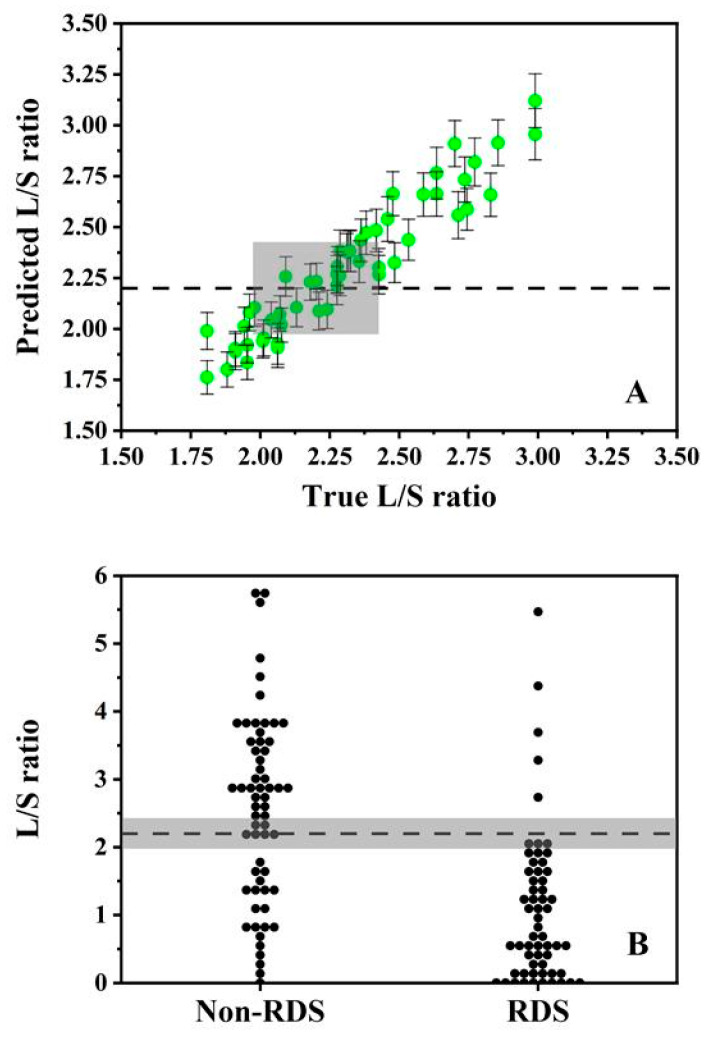
Diagnostic grey zone analysis of the water-based complex mixture models. The L/S ratio region is zoomed in from 1.50 to 3.50, with the diagnostic threshold set at 2.2 indicated by the black dashed lines. Grey zones were defined by prediction intervals of ±0.23 for the water-based model (**A**). Application to published reference L/S ratios shows the distribution of non-RDS and RDS cases within and outside the grey zone under the assumed uncertainty model (**B**). Data points outside the viewing frame are not displayed but were included in the analysis. Clinical data reproduced with permission from Verder et al. [22].

The diagnostic relevance of these uncertainty estimates is most clearly illustrated for the derived L/S ratio and the commonly cited threshold of 2.2. A diagnostic ‘grey zone’ was defined as predictions whose 95% prediction interval encompassed 2.2, representing cases that cannot be assigned with confidence to either side of the threshold under the present uncertainty model. Using the averaged test-set uncertainty for the L/S predictions, this corresponded to a grey-zone half-width of ±0.23 (Figure 6A), such that a true value of 2.2 would be expected to yield predicted values within the range of 1.97–2.43 in 95% of cases. To illustrate the clinical decision implications of this uncertainty model, the grey-zone decision rule was applied to the published reference L/S ratios from a neonatal cohort (*n* = 133; 74 non-RDS, 59 RDS) [22,24]. This analysis does not constitute validation of the spectral model on patient spectra; rather, it projects the expected impact of the uncertainty model assuming unbiased L/S estimates and the same uncertainty structure. Under this decision rule, six non-RDS and six RDS infants fall within the indeterminate region (~9%) (Figure 6B). Under an intention-to-diagnose rule, in which grey-zone outcomes are treated as test failures, the projected outcomes correspond to 48 true positives, 11 false negatives, 48 true negatives and 26 false positives, yielding an effective sensitivity of 81% and specificity of 65%.

From a methodological perspective, the narrow prediction intervals are consistent with the repeatability findings in Section 3.1, where scan-to-scan variability was negligible and the dominant uncertainty was attributable to film preparation. Adoption of the AQ-dry protocol reduces preparation-driven dispersion, which in turn reduces the spread of plausible predictions for a given spectrum. From a clinical perspective, the grey-zone decision rule provides a decision-relevant interpretation of uncertainty: rather than forcing a binary classification near the threshold, it explicitly identifies borderline cases for which additional clinical information or repeat testing may be required. Further reductions in the indeterminate fraction are expected with increased training density around the threshold region and, critically, with model development using patient-derived samples, where matrix effects and biological variability should be incorporated into both model calibration and interval estimation. Because the PLSR models were trained on synthetic mixtures, these intervals quantify analytical uncertainty within the controlled calibration domain; extension to patient-derived spectra will require calibration that captures clinical matrix variability, consistent with the run-dominated variance structure observed for the ETA extract in Section 3.1.

**Figure 7:**
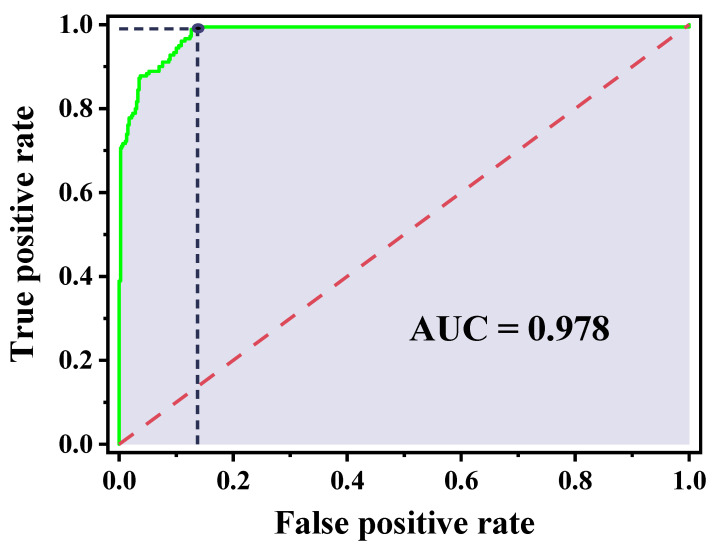
ROC curve for L/S classification using the synthetic mixture dataset. Binary reference labels were defined from the true L/S ratios relative to the clinical threshold of 2.2, and the predicted L/S point estimate was used as the continuous score. The green trace shows the ROC curve (AUC = 0.978), with the grey shaded region representing the AUC. The red dashed diagonal indicates chance-level discrimination. The marked operating point illustrates a rule-out-oriented setting, achieving true positive rate = 0.990 at false positive rate = 0.137, with the black dashed lines indicating the corresponding coordinates on the axes.

To provide a threshold-independent measure of discrimination that is distinct from this uncertainty-driven grey-zone decision rule, ROC analysis was additionally performed using the synthetic mixture dataset, with binary reference labels defined by the true L/S ratios relative to 2.2 and the predicted L/S ratios estimate used as the continuous score; this yielded an AUC of 0.978, indicating excellent discrimination across operating points (Figure 7). Where sensitivity is prioritised (rule-out-oriented use), an operating point achieving a true positive rate = 0.990 (sensitivity = 0.990) corresponds to a false positive rate = 0.137 (specificity = 0.863). Taken together, the ROC curve summarises the ranking performance of the synthetic model’s predictions under forced binary classification, whereas the grey-zone decision rule makes explicit how prediction uncertainty around the clinical threshold can translate into indeterminate outcomes and reduced effective sensitivity/specificity when applied in a decision context. As this ROC analysis is based on synthetic mixtures with reference labels derived from true L/S values, performance may not directly translate to clinical matrices, where biological variability and sampling effects can broaden class overlap.

## 4. Conclusions

This study evaluated the effect on ATR-FTIR spectra of films generated from synthetic and extracted lipids from human ETA films, produced using two distinct drying routes. It was found that samples prepared using an AQ-dry route had a significantly higher SNR and less run-to-run variance compared to the DCM-dry route. Variance decomposition analysis showed that the preparation route dominated the spectra, with the nested run-to-run variability contributing most of the remaining variance. This provides evidence towards variability in film morphology rather than an instrument or environmental limitation. Based on this, we demonstrated a PLSR model generated using synthetic lipid mixtures that reduces the uncertainty-limited diagnostic grey zone to L/S +/− 0.23 moles/mole for a previously reported neonatal clinical cohort.

These observations define a practical sample presentation requirement for translation to a point-of-care nRDS test. Since sample preparation governs measurement reliability, sample handing must be standardised to guarantee bedside performance. This can be achieved through automation or integration of the lipid extraction into a microfluidic workflow to ensure repeatable film formation and safe clinical use. An alternative implementation would be to minimally process and measure the samples directly, reducing device complexity and per-test cost but requires more sophisticated machine learning interpretation to mitigate the presence of additional surfactant components.

Overall, these findings demonstrate that controlling preparation-dependent film formation is central to improving spectral quality, repeatability, and uncertainty-aware interpretation in dried-film ATR-FTIR surfactant lipid analysis. Future work will focus on extending the workflow to patient-derived samples and incorporating our finding on clinically relevant matrix effects into model calibration and uncertainty estimation.

## Figures and Tables

**Table 1:** Selected wavenumber regions associated with certain vibrational bands in tested surfactant lipids [39,40,41,42,43,44,45,46].

Wavenumber (cm^−1^)	Vibrational Band
2930–2920	C–H antisymmetric stretching [39,40]
2860–2850	C–H symmetric stretching [39,40]
1745–1735	C=O stretching [39,40]
1650–1645	C=C stretching [41], C=O stretching [42]
1475–1465	C–H bending [40,42]
1260–1240	PO_2_^−^ asymmetric stretching [43]
1095–1090	P–O–R symmetric stretching [40,41,45]
1070–1060	P–O–R stretching [41], C–O–C stretching [40,42]
975–965	N^+^(CH_3_)_3_ and O–CH_3_ groups stretching [40,42,46], C=C bending [44]

**Table 2:** The 2920 cm^−1^ peak SNR for DCM-dry and AQ-dry preparations. Values are reported as mean ± standard deviation (SD).

Sample Type	2920 cm^−1^ Peak SNR	
	DCM-Dry	AQ-Dry
DPPC	45.187 ± 1.553	71.284 ± 0.846
POPC	20.405 ± 1.780	20.548 ± 0.346
S	6.810 ± 0.462	102.326 ± 1.223
PG	0.975 ± 0.136	40.872 ± 0.699
Chol	3.679 ± 0.378	0.062 ± 0.005
Mid-point concentration mixture	20.128 ± 0.221	128.200 ± 0.621
ETA lipid extract	6.334 ± 0.127	8.134 ± 0.442

**Table 3:** Two-factor mixed nested ANOVA and variance-component decomposition for the mid-point concentration mixture (2920 cm^−1^ peak height), with drying route (DCM-dry vs. AQ-dry) treated as a fixed factor and sample run treated as a random factor nested within route. σ^2^ denotes the estimated variance component attributed to each source, and variance fraction (%) denotes the proportion of total variance attributable to that source. ‘Corrected total’ is reported for completeness and represents the overall variability in the dataset; the corresponding total variance component equals the sum of the variance components listed above (within rounding).

Source	Degrees of Freedom	Mean Squares	F	*p*	σ^2^	Variance Fraction (%)
Drying route	1	3.579	77.857	1.521 × 10^−7^	0.044	89.52
Sample run (Drying route)	16	0.046	692,177	4.9 × 10^−324^	0.005	10.48
Residuals	144	6.642 × 10^−8^			6.642 × 10^−8^	1.36 × 10^−4^
Corrected total	161	0.027			0.049	

**Table 4:** Two-factor mixed nested ANOVA and variance component decomposition for an extracted lipid sample prepared from human ETA (2920 cm^−1^ peak height), with drying route (DCM-dry vs. AQ-dry) treated as a fixed factor and sample run treated as a random factor nested within the route. σ^2^ denotes the estimated variance component attributed to each source, and variance fraction (%) denotes the proportion of total variance attributable to that source. ‘Corrected total’ is reported for completeness and represents the overall variability in the dataset; the corresponding total variance component equals the sum of the variance components listed above (within rounding).

Source	Degrees of Freedom	Mean Squares	F	*p*	σ^2^	Variance Fraction (%)
Drying route	1	0.003	0.523	0.480	0	0
Sample run (Drying route)	16	0.007	29,603	1.66 × 10^−244^	7.327 × 10^−4^	99.97
Residuals	144	2.228 × 10^−7^			2.228 × 10^−7^	0.03
Corrected total	161	6.770 × 10^−4^			7.329 × 10^−4^	

**Table 5:** Prediction interval summary for the water-based lipid prediction models generated using the jackknife+ -after-bootstrap method. ‘+’ and ‘−’ indicate maximum upper prediction intervals and maximum lower prediction intervals, respectively.

Lipid Type	Maximum Prediction Intervals (mM)
DPPC	+0.081, −0.082
POPC	+0.077, −0.072
S	+0.086, −0.074
PG	+0.071, −0.078
Chol	+0.031, −0.027
L	+0.099, −0.082

## Data Availability

All data supporting this study are available from the University of Southampton repository at: https://eprints.soton.ac.uk/ (accessed on 6 February 2026).

## Supplementary Materials

The following supporting information can be downloaded at: https://www.mdpi.com/article/10.3390/bios16030154/s1, Table S1: The 2945–2880 cm^−1^ peak area SNR for DCM-dry and AQ-dry preparations; Table S2: The 1740 cm^−1^ peak SNR for DCM-dry and AQ-dry preparations; Table S3: Two-factor mixed nested ANOVA and variance component decomposition for the mid-point concentration mixture (2945–2880 cm^−1^ peak area), with drying route (DCM-dry vs. AQ-dry) treated as a fixed factor and sample run treated as a random factor nested within route; Table S4: Two-factor mixed nested ANOVA and variance component decomposition for an extracted lipid sample prepared from human ETA (2945–2880 cm^−1^ peak area), with drying route (DCM-dry vs. AQ-dry) treated as a fixed factor and sample run treated as a random factor nested within route; Table S5: Two-factor mixed nested ANOVA and variance component decomposition for the mid-point concentration mixture (1740 cm^−1^ peak height), with drying route (DCM-dry vs. AQ-dry) treated as a fixed factor and sample run treated as a random factor nested within route; Table S6: Two-factor mixed nested ANOVA and variance component decomposition for an extracted lipid sample prepared from human ETA (1740 cm^−1^ peak height), with drying route (DCM-dry vs. AQ-dry) treated as a fixed factor and sample run treated as a random factor nested within route.

## Abbreviations

The following abbreviations are used in this manuscript:

ANOVA	Analysis of variance
aq	Aqueous
ATR	Attenuated total reflectance
AUC	Area under the curve
BAL	Bronchoalveolar lavage
Chol	Cholesterol
DCM	Dichloromethane
DPPC	Dipalmitoylphosphatidylcholine
ELISA	Enzyme-linked immunosorbent assay
ETA	Endotracheal aspirate
FTIR	Fourier transform infrared
L	Lecithin
L/S	Lecithin-to-sphingomyelin ratio
MCT	Mercury cadmium telluride
MSE	Mean square error
nRDS	Neonatal respiratory distress syndrome
PC	Principal component
PG	Phosphatidylglycerol
PLSR	Partial least squares regression
POPC	1-palmitoyl-2-oleoylphosphatidylcholine
R^2^	Coefficient of determination
RDS	Respiratory distress syndrome
RMS	Root mean square
RMSE	Root mean square error
ROC	Receiver operating characteristic
RSD	Relative standard deviation
S	Sphingomyelin
SD	Standard deviation
SNR	Signal-to-noise ratio
