# Supplementary material for: Effects of Sample Deposition Medium and Drying on Spectroscopic Quantification of Lipid Biomarkers in Respiratory Distress Syndrome
Source: Biosensors (Basel). 2026 Mar 10;16(3):154. doi: 10.3390/bios16030154 (PMC13024144; doi:10.3390/bios16030154)
Supplement: Supplementary file 1 [file biosensors-16-00154-s001.zip › biosensors-4173315-supplementary.pdf]

biosensors

MDPI

Supplementary Materials

Effects of Sample Deposition Medium and Drying on Spectroscopic Quantification of Lipid Biomarkers in Respiratory Distress Syndrome

Zixing (Hings) Luo [1,2,†], Waseem Ahmed [1,2,†], Anthony D. Postle [2,3], Ahilanandan Dushianthan [2,3,4], Michael P. W. Grocott [2,3,4] and Ganapathy Senthil Murugan [1,*]

1 Optoelectronics Research Centre, University of Southampton, Southampton SO17 1BJ, UK; hings.luo@soton.ac.uk (Z.L.); waseem.ahmed@soton.ac.uk (W.A.)
2 Perioperative and Critical Care Theme, NIHR Southampton Biomedical Research Centre, University Hospital Southampton NHS Foundation Trust, Southampton SO16 6YD, UK; a.d.postle@soton.ac.uk (A.D.P.); a.dushianthan@soton.ac.uk (A.D.); mike.grocott@soton.ac.uk (M.P.W.G.)
3 Clinical and Experimental Sciences, Faculty of Medicine, University Hospital Southampton NHS Foundation Trust, Southampton SO16 6YD, UK
4 General Intensive Care Unit, University Hospital Southampton NHS Foundation Trust, Southampton SO16 6YD, UK
* Correspondence: smg@orc.soton.ac.uk
† These authors contributed equally to this work.

Supplementary Table S1

Table S1. 2945-2880 cm^{-1} peak area SNR for DCM-dry and AQ-dry preparations. Values are reported as mean ± SD.

Sample type	2945-2880 cm^{-1} peak area SNR	
	**DCM-dry**	**AQ-dry**
DPPC	1329.816 ± 45.185	2082.564 ± 24.874
POPC	685.179 ± 61.302	697.212 ± 11.659
S	191.100 ± 13.273	2955.240 ± 34.682
PG	26.384 ± 4.097	1188.055 ± 20.220
Chol	249.453 ± 26.000	4060.346 ± 221.694
Mid-point concentration mixture	984.070 ± 11.294	3572.190 ± 17.272
ETA lipid extract	261.255 ± 7.826	300.413 ± 15.745

Supplementary Table S2

Table S2. 1740 cm^{-1} peak SNR for DCM-dry and AQ-dry preparations. Values are reported as mean ± SD.

Sample type	1740 cm^{-1} peak SNR	
	**DCM-dry**	**AQ-dry**
DPPC	23.993 ± 0.835	38.897 ± 0.451
POPC	13.079 ± 1.094	13.880 ± 0.236

S	0.021 ± 0.013	0.541 ± 0.014
PG	0.441 ± 0.056	20.103 ± 0.349
Chol	0.038 ± 0.007	0.010 ± 0.001
Mid-point concentration mixture	10.494 ± 0.095	46.381 ± 0.219
ETA lipid extract	1.644 ± 0.026	2.164 ± 0.118

Supplementary Table S3

Table S3. Two-factor mixed nested ANOVA and variance-component decomposition for the mid-point concentration mixture (2945-2880 cm^{-1} peak area), with drying route (DCM-dry vs AQ-dry) treated as a fixed factor and sample run treated as a random factor nested within route. σ^2 denotes the estimated variance component attributed to each source, and variance fraction (%) denotes the proportion of total variance attributable to that source. 'Corrected total' is reported for completeness and represents the overall variability in the dataset; the corresponding total variance component equals the sum of the variance components listed above (within rounding).

Source	Degrees of freedom	Mean squares	F	p	σ^2	Variance fraction (%)
Drying route	1	4177.003	71.323	2.727×10^{-7}	50.845	88.65
Sample run (Drying route)	16	58.565	1087575	$<1\times10^{-1000}$	6.507	11.35
Residuals	144	5.385×10^{-5}			5.385×10^{-5}	9.389×10^{-5}
Corrected total	161	31.764			57.352	

Supplementary Table S4

Table S4. Two-factor mixed nested ANOVA and variance-component decomposition for an extracted lipid sample prepared from human ETA (2945-2880 cm^{-1} peak area), with drying route (DCM-dry vs AQ-dry) treated as a fixed factor and sample run treated as a random factor nested within route. σ^2 denotes the estimated variance component attributed to each source, and variance fraction (%) denotes the proportion of total variance attributable to that source. 'Corrected total' is reported for completeness and represents the overall variability in the dataset; the corresponding total variance component equals the sum of the variance components listed above (within rounding).

Source	Degrees of freedom	Mean squares	F	p	σ^2	Variance fraction (%)
Drying route	1	7.271	0.748	0.400	0	0
Sample run (Drying route)	16	9.719	3849	8.768×10^{-181}	1.080	99.77
Residuals	144	0.003			0.003	0.23
Corrected total	161	1.013			1.083	

https://doi.org/10.3390/bios16030154

Supplementary Table S5

Table S5. Two-factor mixed nested ANOVA and variance-component decomposition for the mid-point concentration mixture (1740 cm^{-1} peak height), with drying route (DCM-dry vs AQ-dry) treated as a fixed factor and sample run treated as a random factor nested within route. σ^2 denotes the estimated variance component attributed to each source, and variance fraction (%) denotes the proportion of total variance attributable to that source. 'Corrected total' is reported for completeness and represents the overall variability in the dataset; the corresponding total variance component equals the sum of the variance components listed above (within rounding).

Source	Degrees of freedom	Mean squares	F	p	σ^2	Variance fraction (%)
Drying route	1	1.279	82.788	1.005×10^{-7}	0.015	90.09
Sample run (Drying route)	16	0.0156	607223	5.835×10^{-339}	0.002	9.91
Residuals	144	2.545×10^{-8}			2.545×10^{-8}	1.469×10^{-4}
Corrected total	161	0.010			0.017	

Supplementary Table S6

Table S6. Two-factor mixed nested ANOVA and variance-component decomposition for an extracted lipid sample prepared from human ETA (1740 cm^{-1} peak height), with drying route (DCM-dry vs AQ-dry) treated as a fixed factor and sample run treated as a random factor nested within route. σ^2 denotes the estimated variance component attributed to each source, and variance fraction (%) denotes the proportion of total variance attributable to that source. 'Corrected total' is reported for completeness and represents the overall variability in the dataset; the corresponding total variance component equals the sum of the variance components listed above (within rounding).

Source	Degrees of freedom	Mean squares	F	p	σ^2	Variance fraction (%)
Drying route	1	2.637×10^{-4}	1.521	0.235	1.114×10^{-6}	5.47
Sample run (Drying route)	16	1.734×10^{-4}	22037	2.800×10^{-235}	1.927×10^{-5}	94.50
Residuals	144	7.869×10^{-9}			7.869×10^{-9}	0.03
Corrected total	161	1.888×10^{-5}			2.039×10^{-5}	

https://doi.org/10.3390/bios16030154
